# Pathophysiological Basis for the Formation of the Tumor Microenvironment

**DOI:** 10.3389/fonc.2016.00066

**Published:** 2016-04-12

**Authors:** Michael R. Horsman, Peter Vaupel

**Affiliations:** ^1^Department of Experimental Clinical Oncology, Aarhus University Hospital, Aarhus, Denmark; ^2^Department of Radiooncology and Radiotherapy, Klinikum rechts der Isar, Technische Universität München (TUM), Munich, Germany

**Keywords:** hypoxia, tumor microenvironment, radiotherapy, chemotherapy, malignant progression

## Abstract

Poor microenvironmental conditions are a characteristic feature of solid tumors. Such conditions occur because the tumor vascular supply, which develops from the normal host vasculature by the process of angiogenesis, is generally inadequate in meeting the oxygen and nutrient demands of the growing tumor mass. Regions of low oxygenation (hypoxia) is believed to be the most critical deficiency, since it has been well documented to play a significant role in influencing the response to conventional radiation and chemotherapy treatments, as well as influencing malignant progression in terms of aggressive growth and recurrence of the primary tumor and its metastatic spread. As a result, significant emphasis has been placed on finding clinically applicable approaches to identify those tumors that contain hypoxia and realistic methods to target this hypoxia. However, most studies consider hypoxia as a single entity, yet we now know that it is multifactorial. Furthermore, hypoxia is often associated with other microenvironmental parameters, such as elevated interstitial fluid pressure, glycolysis, low pH, and reduced bioenergetic status, and these can also influence the effects of hypoxia. Here, we review the various aspects of hypoxia, but also discuss the role of the other microenvironmental parameters associated with hypoxia.

## Introduction

Most solid tumors are just like normal tissues in that they need a regular supply of oxygen and nutrients to be able to exist, as well as processes for the elimination of the waste products of cellular metabolism. When tumors first appear, this function is provided by the normal blood supply of the host organ in which the tumor arises. However, unlike normal tissues, tumors continually expand in size and a point is reached where the host vascular supply becomes inadequate in supplying these needs. To compensate, tumors will actually develop their own functional vascular supply. This they do from the normal host vessels by the process of angiogenesis. Unfortunately, the tumor neo-vasculature that is formed is not only primitive and chaotic when compared to the normal tissue vascular supply from which it develops, but it also suffers from numerous structural and functional abnormalities. As a result, it is still unable to meet all the demands of the growing tumor mass (with sizes larger than 2–3 mm). Consequently, a hostile microenvironment develops within the tumor and this can be summarized by the so-called “crucial Ps.” These are listed in Table 1 and basically reflect conditions of poor perfusion, oxygen deprivation, nutrient deficiency, severe acidity, and elevated interstitial fluid pressure (IFP).

**Table 1:** **The crucial Ps characterizing the hostile tumor microenvironment**.

**Pathophysiological characteristics showing a reduced level**
• Partial pressure of oxygen
• Production of high-energy compounds
• pH of the extracellular compartment
• Paucity of nutrients
• Paucity of bicarbonate
**Pathophysiological characteristics showing an enhanced level**
• Perfusion inadequacies/vascular chaos
• Perfusion heterogeneities
• Permeability of tumor microvessels
• Pressure of interstitial fluid
• Production of lactate
• Production of protons
• Production of adenosine
• Partial pressure of carbon dioxide

*Modified from Ref. (1)*.

The tumor cells that exist in this hostile microenvironment are actually still viable. But, as a result of being in these adverse microenvironmental conditions, those same tumor cells can exhibit resistance to conventional cancer therapies, including radiation and certain types of chemotherapy. The poor tumor microenvironment also causes these cells to upregulate the expression of various genes and biosynthesis of proteins, an effect that not only increases their survival potential but can also increase tumor aggressiveness and metastatic spread. Numerous attempts have, and are being made, to identify the microenvironmental conditions within tumors so as to select appropriate therapies to target those cancer cells that thrive in the hostile microenvironment.

Of these poor microenvironmental conditions within tumors, low oxygenation (hypoxia) is the one that has been the focus of most studies and is often considered as the only factor of importance. While hypoxia is clearly critical for outcome of cancer patients, it is generally associated with the other crucial Ps and as such any discussion of the role of hypoxia in tumors must be made in connection with the other hostile microenvironmental parameters. Thus, we will review the general pathophysiological characteristics of the tumor microenvironment, how that microenvironment develops, and what significance that has for cancer.

## Factors Influencing the Tumor Microenvironment

### Importance of the Tumor Vascular Supply

Angiogenesis is clearly an essential requirement for the growth and development of solid tumors (2–4). This process begins with the release of angiogenic factors, primarily vascular endothelial growth factor (VEGF), by the tumor cells (5). The actual triggers that initiate this process are not fully established. Loss of suppressor gene function and oncogene activation certainly play a role (5, 6), but the development of hypoxia as a result of tumor growth is also a major factor (6). Additional studies with tumor cells grown in culture have shown that the secretion rate of the VEGF protein increases as soon as the oxygen concentration is lowered from 21 to only 5% and that this secretion rate increases as the oxygen concentration decreases reaching maximal levels at around 0.5% and below (7). Release of VEGF and other growth factors set in motion a number of biochemical and physical steps that include enzymatic destruction of the basal membrane of the endothelial cells of the host vasculature, migration of endothelial cells into the extracellular matrix to form sprouts, and endothelial cell division away from the sprout tip (8). Solid strands of endothelial cells are then formed in the extracellular matrix, a lumen develops within those strands, neighboring sprouts fuse to form loops, and from the primary loops new buds and sprouts emerge (8). Finally, functional vessels are established.

**Figure 1:**
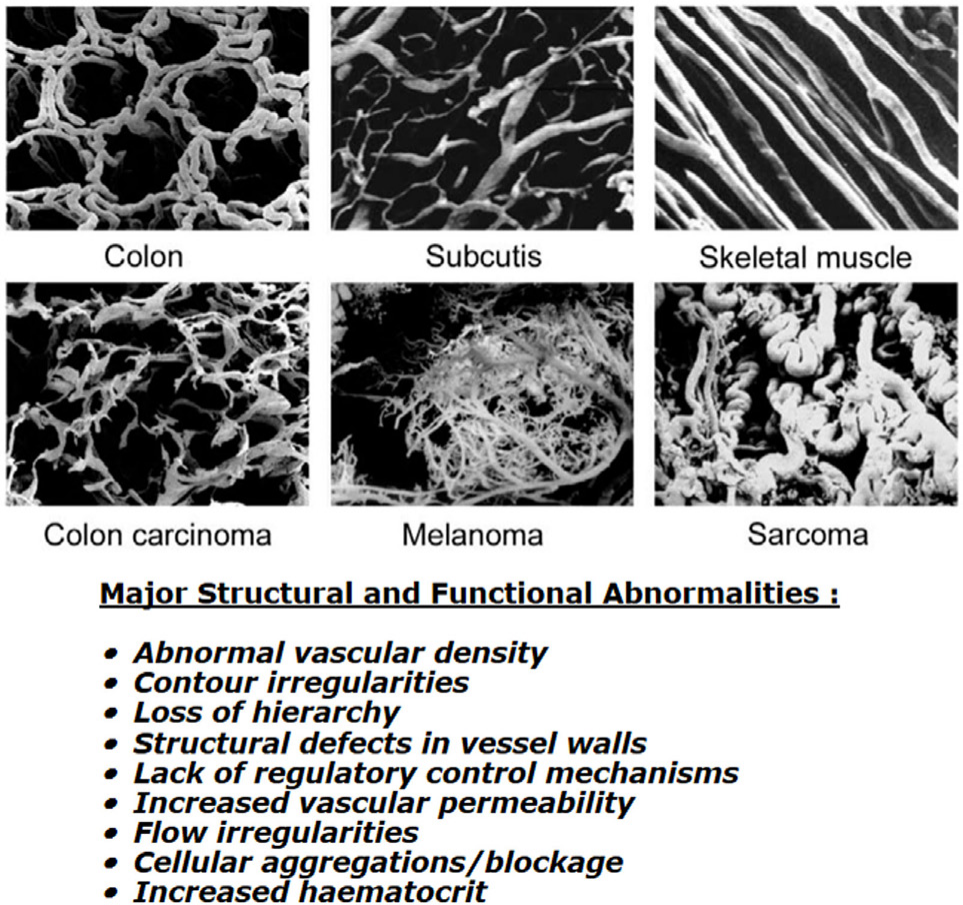
**Vascular casting images showing differences in microcirculation between normal tissues (top three panels) and malignant tumors (lower three panels)**. Specific details of the corrosion casting technique used to produce these images can be found in Konerding et al. (14). Images shown were obtained courtesy of Prof. Konerding, Dept. Functional and Clinical Anatomy, University Medical Center, Mainz, Germany and are from Vaupel (10). Bottom text lists the major structural and functional abnormalities of tumor vessels when compared to normal tissues; composite information based on work by Kimura et al. (12), Reinhold and van der Berg-Blok (15), Vaupel et al. (10, 16), and Baronzio et al. (17).

Although a functional tumor vascular supply is necessary, the neo-vasculature that develops is actually inadequate to meet all the demands of the growing tumor mass. Endothelial cells divide at a slower rate than tumor cells (9) and as a consequence, the developing tumor vasculature is unable to keep pace with the expanding tumor population. The tumor vasculature that is formed is also very different from that of normal tissues (see Figure 1). Structurally, it is very chaotic. Vascular density is abnormal with increased intervessel distances and the existence of avascular areas. There are contour irregularities reflected by vessels that are elongated, tortuous, and large, and have aberrant branching and blind ends. The pattern of vessel interconnection is also haphazard. Unlike normal vessels, there is a loss of hierarchy. The vessels are also very primitive in nature, having incomplete or missing basement membranes and endothelial lining, and lacking pericytes, smooth muscle, pharmacological receptors, and even innervation. Tumor vessels are often highly permeable allowing significant plasma leakage. Due to an absence of vasomotion and flow regulation, blood flow velocities through tumor vessels can be unstable as can the direction of flow [for review, see Ref. (10)]. It has been estimated that 1–8% of vessels can experience flow stasis (11, 12) and that around 8% of all microvessels show plasma flow only (12). Some of these changes will be mediated through effects of the various blood-borne cells. These include erythrocyte sludging, leukocyte sticking, and blockage of vessels by circulating white blood cells or tumor cells. High IFP can also play a role here. In normal tissues, there exists a perfusion pressure difference of about 20 mmHg between the arterial and venous ends of microvessels and this drives blood flow through capillaries. However, in tumors, the increased leakiness of vessels and the lacking of functional lymphatics result in an increased IFP. Transmural coupling between this high IFP and microvascular pressures can result in abolished perfusion pressure differences between the arterial and venous ends, thus, causing flow stasis and hypoxia (13). However, this is more likely to result in chronic rather than acute hypoxia (13). Finally, the hematocrit within tumor microvessels can be increased by 5–14% and this will also influence flow.

### Other Parameters Affecting the Micromilieu

Apart from the inadequacies of the tumor vascular supply, there are a number of other important factors that can influence the microenvironment within tumors. Chief among these is the oxygen carrying capacity of the blood. This can be substantially reduced under specific conditions, thus making less oxygen available. Such an effect is seen with anemia where the normal hemoglobin levels of 7.5–9.5 mmol/L in females and 8–10 mmol/L in males can be reduced by 50% in anemic patients. Additional studies in which tumor oxygenation status was directly measured with polarographic needle electrodes have reported a correlation between the level of tumor hypoxia and hemoglobin concentration (18). Reduced oxygen availability is also observed in patients who smoke. Smoking impairs the delivery of oxygen to tumors due to the presence of carboxyhemoglobin (HbCO) that is formed by the binding of carbon monoxide (CO) to hemoglobin (19). Heavy smokers can have up to 16–18% HbCO in their blood. This reaction not only decreases the amount of hemoglobin available for oxygen transport but will also shift the oxygen-dissociation curve to the left making it more difficult for the hemoglobin to release oxygen to the cells. Since the affinity of hemoglobin for CO is approximately 250 times the affinity for oxygen, even low concentrations of CO can result in significant levels of HbCO in the blood.

The microenvironment of tumors will also depend on the cellular consumption of oxygen and essential nutrients. As a result of tumor cells close to the vascular supply consuming what they need for growth and survival, less will be available for those cells further away. Consequently, radial oxygen, nutrient, and pH gradients are established (16). The extent of those gradients will depend not only on the rate of consumption but also on what is actually delivered to the cells by the blood supply. Indeed, it has been reported that in the case of oxygen the cells next to the blood vessel can have oxygen concentrations as low as 2% [approximately 15 mmHg; (20)] and this would certainly reduce the oxygen diffusion distance. As it is the case in normal physiology, there is also an intravascular (longitudinal) oxygen partial pressure gradient when the blood moves from the arterial to venous end of the microvessels (21). All these factors, coupled with the structural and functional aberrations described in the previous section, will result in the development of areas within the tumor that can be considered “abnormal and adverse” when compared to those conditions found in normal tissues (10, 16).

## Microenvironmental Characteristics of Tumors

### Hypoxia

**Figure 2:**
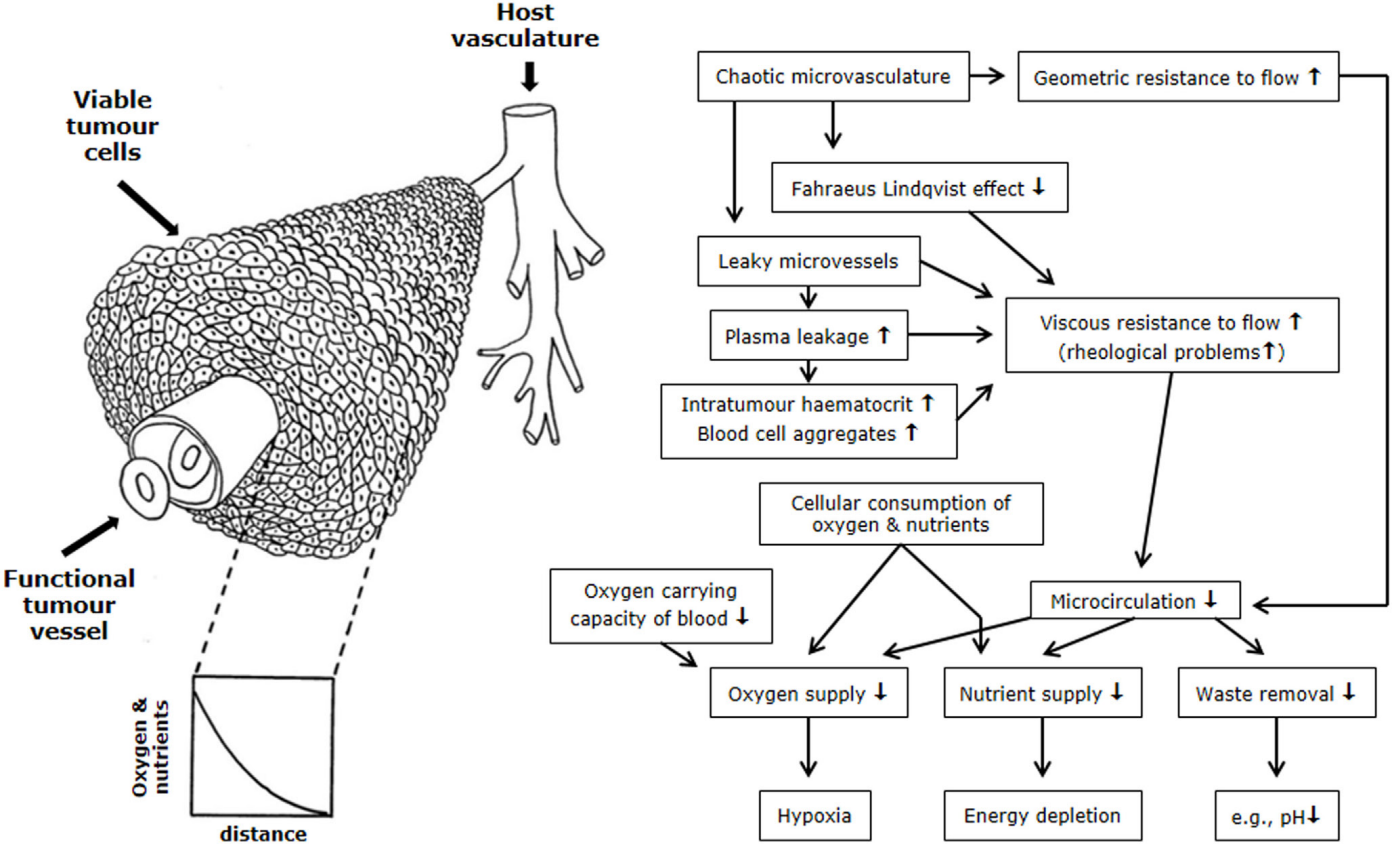
**Schematic illustration of the relationship between the tumor vasculature and microenvironment**. The left side shows tumor cells growing in a corded structure around a functional vessel from which the cells receive their oxygen and nutrient supply, but as these substances diffuse out from the vessel they are utilized by the cells so that gradients are established. On the right side is a flow chart showing the relationship between the hostile microenvironment of tumors and the factors that give rise to its development. Figure is modified from Ref. (1, 27).

The microenvironmental parameter that has been the most extensively investigated is hypoxia. By definition, hypoxia is a state of reduced oxygenation that influences biological functions (22). The first indirect indication that hypoxia existed in tumors was made by Thomlinson and Gray (23). From histological sections of carcinomas of the bronchus, they typically found viable tumor regions surrounded by vascular stroma, with regions of necrosis evolving in the center of the tumor mass. The thickness of the resulting shell of viable tissue was found to be between 100 and 180 μm. They suggested that as oxygen diffused from the stroma, it was consumed by the cells, and although those beyond the diffusion distance were unable to survive, the cells immediately bordering the necrosis might be viable yet hypoxic; unfortunately, in this concept, the diffusion of glucose and other nutrients is completely ignored. Later, an inverted version of the Thomlinson and Gray model was described, with functional blood vessels surrounded by cords of viable tumor cells outside of which were areas of necrosis [Krogh model of oxygen diffusion; (24)]. This corded structure is the more typical picture found in most solid tumors and is illustrated in Figure 2. As with the Thomlinson and Gray model, an oxygen gradient is created as the oxygen diffuses from the blood vessel, resulting in a region of cells at the edge of the cord that are oxygen deprived and are commonly referred to as diffusion-limited chronic hypoxia. This type of hypoxia has been seen in both animal and human tumors (25). It has been suggested that such hypoxic cells can survive under these adverse conditions for several days (26). Death will also occur as the hypoxic cells move further away from the blood supply as the tumor grows, although this is more likely to result from a glucose deficit rather than just a lack of oxygen.

Diffusion-limited chronic hypoxia was the working model for hypoxia from the 1950s until around the 1980s when it was then suggested that a second type of hypoxia could exist in tumors and one that was acute in nature (28). This was later confirmed and shown to be the result of the transient stoppages in tumor blood flow described earlier (29). The hypoxia that results was originally called perfusion limited acute hypoxia, although other terms are often used, including cyclic, intermittent, transient, repetitive, or fluctuating, and it is probably the latter term that is the most appropriate description. Evidence for fluctuating hypoxia has been reported in murine tumors (30), human tumor xeongrafts (31), and even in tumors in cancer patients (32–34).

Today, hypoxia is often considered as a single entity even though we know there are at least two types. However, even the concept of chronic and acute/fluctuating hypoxia is an over-simplification of the real picture (13). Acute hypoxia can result from a total or partial shut-down in perfusion (12); a complete shut-down would starve cells of oxygen and nutrients and result in ischemic hypoxia, which would not be the case for a partial shut-down where plasma flow, thus nutrient supply, can occur leading to hypoxemic hypoxia. For chronic hypoxia, the picture is even more complicated. It can result from a diffusion limitation under “normal” conditions (diffusional hypoxia), or be due to reduced oxygen availability as with high HbCO or anemia (anemic hypoxia). But, it will also be dependent on the oxygenation level; cells close to the vessel could be slightly hypoxic, while cells next to necrosis could even be anoxic (a situation where no oxygen can be detected) but still viable if they have sufficient nutrient supply (i.e., glucose). Whatever the description, hypoxia is now known to be a characteristic feature of most solid animal tumor models (35) and numerous human cancers (16, 36).

### Interstitial Fluid Pressure

Unlike normal tissues, tumors often contain vessels that are abnormally leaky and also lack a functional lymphatic system (37). These, coupled with a large hydraulic conductivity, results in a significant bulk flow of free fluid in the interstitial space. In normal tissues, water influx into the interstitial compartment has been estimated to be between 0.5 and 1.0% of plasma flow, yet in human tumors values up to 15% can be reached (38). As a result of fluid accumulating in the tumor matrix, there is a build-up of interstitial pressure (39–41). Interstitial fibrosis, contraction of the interstitial space mediated by stromal fibroblasts, and high oncotic pressures within the interstitium may also contribute to the development of interstitial hypertension (42). In most normal tissues, IFP is just above or below atmospheric values (43), but in tumors it can reach 50 or even 100 mmHg (1). IFP is generally uniformally high throughout the center of tumors, but drops steeply in the tumor periphery (44). However, since vascular permeability varies from tumor to tumor and can be heterogeneous within the same tumor, IFP is not constant (1). It can also fluctuate with changes in microvascular pressures (45).

### Glycolysis and pH

Warburg’s classic work in the 1920s showed that cancer cells intensively converted glucose to lactate (glycolysis) even in the presence of oxygen [for a review see Ref. (46)]. Today, it is believed that there is no clear evidence that cancer cells are inherently glycolytic, but that some tumors might be glycolytic *in vivo* as a result of hypoxic response mechanisms (47). Hypoxia will shift the balance of cellular energy production toward glycolysis with the generation and subsequent accumulation of lactate (48). Indeed, several studies have found high median lactate levels of around 7 mM in head and neck cancers (49) and up to 14 mM in uterine cervix (50). Although lactate is generally considered a waste product, there is evidence that the lactate produced by hypoxic cells can be taken up in normoxic cancer cells via the monocarboxylate transporter-1 and can then be utilized for oxidative phosphorylation instead of glucose as a substrate (51). However, cellular lactate production and release will lead to tumor acidosis. What is clear is that like normal cells, tumor cells have efficient mechanisms for exporting protons into the extracellular space (52, 53), thus a pH gradient exists across the tumor cell membrane so that intracellular pH (pHi) remains higher than the extracellular pH (pHe). In normal tissues, this gradient is reversed such that pHi is actually lower than pHe (16, 54–56). The production and release of lactate alone does not fully account for the acidosis found in the extracellular compartment of solid tumors. Other key mechanisms may play an important role, especially ATP hydrolysis, glutaminolysis, carbon dioxide production, and bicarbonate depletion (48).

### Bioenergetic Status

Various techniques have been used to monitor the bioenergetic status within tumors. These include *ex vivo* quantitative bioluminescence (57) and high-performance liquid chromatography [HPLC; (38, 58)], and non-invasive ^31^P-nuclear magnetic resonance/spectroscopy [NMR/MRS; (59)]. The global concentrations of ATP measured in experimental tumors using HPLC were found to be typically between 0.4 and 2.0 mM (38, 58). These global ATP concentrations and adenylate energy charge only changed marginally provided tumors did not exceed biologically relevant tumor sizes (i.e., 1% of the body weight). With increasing tumor mass, ATP hydrolysis increased. As a result of this increased ATP degradation, an accumulation of purine catabolites, and the final degradation product uric acid, has been observed (38). Using quantitative bioluminescence, the microregional distribution of ATP has been assessed in cryobiopsies of cervix tumors and found to be heterogeneous and comparable to high flow experimental tumors (38). This ATP distribution profile was similar to those seen for both glucose and lactate, but there was no clear-cut correlation between tumor oxygenation and regional ATP levels (38). Bioluminescence measurements of regional ATP distributions in experimental brain tumors reported ATP levels that were similar to normal brain, whereas glucose was slightly lower and lactate substantially higher (38, 60), with these metabolites showing marked tumor heterogeneity (60). Additional studies using NMR have shown that in many human malignancies, high concentrations of phosphomonoesters, phosphodiesters, and inorganic phosphate, as well as low phosphocreatine, are often found. The exception is again in human brain tumors, where no significant differences in ^31^P-NMR spectra were seen when compared to normal brain tissue (38).

### Hypoxia-Driven Adenosine Accumulation

The development of tumor hypoxia is accompanied by a substantial accumulation of the nucleoside adenosine (ADO) in the range of 50–100 μM (61). By contrast, ADO levels in normal tissues have been found to be in the range of 10–100 nM (62, 63). ADO accumulation is preferentially caused by an ATP release from cancer cells into the extracellular space upon hypoxic stress. After transport out of cancer cells, extracellular ATP is converted into ADO by hypoxia/hypoxia-inducible factor (HIF)-sensitive, membrane-bound ectoenzymes CD39 and CD73. Intracellular ADO-formation from AMP by a cytosolic AMP-nucleotidase with subsequent ADO-export into the extracellular space through a nucleoside transporter seems to play a subordinate role. ADO-actions (adenosinergic effects) are mediated upon binding to surface receptors, mainly A2A-receptors on tumor and immune cells. Receptor activation leads to a broad spectrum of strong immune-suppressive properties through modulation of the innate and adaptive immune system, thus facilitating tumor escape from immune control (62, 64–66). Mechanisms include (a) an impaired activity of CD4^+^ T and CD8^+^ T, NK cells, and dendritic cells (DCs), a decreased production of immune-stimulatory lymphokines, and (b) an activation of Treg cells, expansion of myeloid-derived suppressor cells (MDSCs), promotion of pro-tumor M2-macrophages, and increased activity of major immune-suppressive cytokines. In addition, ADO can directly stimulate tumor cell proliferation and angiogenesis. Taken together, there is clear evidence that ADO-mechanisms described can thwart anti-tumor immune responses elecited by radiotherapy and fever-range hyperthermia (67).

## Significance of the Tumor Microenvironment for Cancer

### Radiation

The potential of microenvironmental parameters to influence outcome to radiotherapy was first suggested from experiments in which the radiation response of skin was markedly decreased if the blood flow to the irradiated area was reduced by compression (68). This was followed by a report that tissues in which blood flow was stimulated by diathermia showed a more prominent response to radiation (69). Further experimental observations led Gray and co-workers to finally postulate the role of oxygen deficiency as a major source of radiation resistance (70). This occurs because oxygen is critical for the response of cells to radiation. The mechanism responsible is generally referred to as the “oxygen-fixation hypothesis” (71). When radiation interacts with the cellular target, which is usually DNA, it results in the production of free radicals. These are produced either directly by the radiation itself or indirectly from other molecules that are affected by radiation and then diffuse sufficiently to reach and damage the DNA target. Since water constitutes around 70% of all mammalian cells, most of the indirect radicals are probably produced from water molecules. In the absence of oxygen or in the presence of hydrogen-donating species (i.e., thiols), the free radicals formed in the DNA can react with hydrogen ions and the target is then chemically restored to its original form. However, if oxygen is present, it will react with the free radical to form a product that undergoes further reaction, ultimately producing a chemical change in the target. The damage is now fixed and can only be removed by enzymatic repair processes.

It has been demonstrated from rapid-mix studies that the oxygen effect occurs only if oxygen is present either during irradiation or within a few milliseconds thereafter (72, 73). The amount of oxygen is also critical. An almost maximum enhancement of radiation is seen with oxygen partial pressures above around 20 mmHg (approximately 3%). Below this partial pressure radiation sensitivity decreases in an oxygen-dependent fashion (71); in the absence of oxygen roughly three times as much radiation is required to kill the same number of cells as seen under normoxic conditions. This effect is generally referred to as the “oxygen enhancement ratio” (OER; ratio of the radiation dose in hypoxia/anoxia to that in air, to give the same biological effect). For radiation of higher energy than X-rays produced by modern radiotherapy units the OER actually decreases (74).

Numerous animal studies have demonstrated that hypoxia in tumors can influence radiation response. Three classical assays have been used (35, 75). They are (a) the clamped clonogenic survival assay, in which tumors are excised after treatment and cell survival measured in culture; (b) the clamped tumor growth delay assay, where measurements are made of the time taken for tumors to reach a specific size after treatment; and (c) the clamped tumor control assay, whereafter the percentage of animals showing local tumor control at a certain time after treatment is recorded. For each technique, it is necessary to produce full radiation dose–response curves under air breathing and fully anoxic (clamped tumor) conditions. The results of such assays not only demonstrate that hypoxia in tumors influences radiation response, but the degree of displacement of the dose–response curves also allows us to calculate the actual percentage of radiobiological cells in the tumor. Using these assays, the hypoxic fractions have been estimated to range from 1% to well over 50% of the viable tumor cells in animal tumors and human tumor xenografts (35).

Demonstrating that hypoxia can influence the radiation response of human tumors is more difficult, since none of the above approaches are applicable to humans. Although numerous other methods have been developed to try and identify hypoxia in human tumors (25, 76, 77), not all have been used to demonstrate the relationship between hypoxia and radiation response. The earliest attempts to do the latter were based on indirect approaches (78), and involved estimates of tumor vascularization, using such endpoints as intercapillary distance, vascular density and the distance from tumor cells to the nearest blood vessel (79–81). All showed that patients with less well vascularized tumors, and presumably more hypoxic, had a poorer outcome to radiation therapy. More direct approaches have used exogenous markers that are injected into the host and bind to regions of tumor hypoxia, or endogenous markers that are genes/proteins upregulated under hypoxia. The former include nitroimidazole or copper-based derivatives, which can be identified immunohistochemically from histological sections or non-invasively using positron emission tomography, SPECT, or magnetic resonance spectroscopy (25). Although such exogenous markers can be used to identify tumor hypoxia and even associated with outcome following radiation therapy in head-and-neck carcinoma patients (82), there has never been a proper radomized trial. The endogenous markers include such factors as carbonic anhydrase IX, GLUT-1, HIF-1, and osteopontin (83–86). These can be measured from biopsy material using protein immunohistochemistry or as gene expression, or proteins identified from blood samples. Although endogenous markers have been correlated with outcome to radiation therapy in some studies, it is not a universal finding (82), which probably reflects the fact that many of these endogenous markers are not hypoxia-specific rather than any indication that hypoxia does not play a role in influencing radiation response.

Probably the most direct method for estimating tumor hypoxia and one that has certainly been used to show the negative influence of such hypoxia on radiation response is the measurement of oxygen partial pressure (pO_2_) distributions with polarographic electrodes. Early attempts to achieve this used “home-made” glass electrodes which were cumbersome, fragile, and only a few pO_2_ values 3–4 mm below the surface of the tumor were possible. Nevertheless, clinical data were obtained in cervix (79) and head and neck (87) that clearly demonstrated a relationship between such oxygenation measurements and outcome to radiation therapy, in that those patients with tumors that were better oxygenated had a significantly superior local response to irradiation.

This whole area was revolutionized with the development of the Eppendorf histography system, which had two distinct improvements. The first was having the oxygen microsensor inside a metal needle and the second the attachment of this needle to a stepping motor that allowed for multiple measurements along the needle track through the tumor. Numerous clinical studies were, thus, undertaken in a variety of human tumor types. The results clearly showed that hypoxia was to be found in virtually all human tumors investigated, although the degree of hypoxia could be variable (16, 36, 88, 89). Probably the most significant finding from these studies was the confirmation that hypoxia influenced outcome to therapy, especially where radiation was given. This has been reported for cervix (90–95), head and neck (96–100), soft tissue sarcomas (101, 102), and prostate (103, 104). Results for all four tumor types are summarized in Table 2 and clearly illustrate that the patients with more hypoxic tumors had a poorer outcome response.

**Table 2:** **Relationship between tumor oxygenation estimated prior to therapy using the Eppendorf histograph and outcome to therapy**.

Tumor type	Patient treatments^a^	No. of patients	Response endpoints^b^	Less hypoxic (%)	More hypoxic (%)	Oxygenation endpoint^c^	Reference
Cervix	RT/CT/SG	31	OS at 22 months	80	32	Median pO_2_ above or below 10 mmHg	(90)
	RT/CT/SG	89	OS at 5 years	69	37	Median pO_2_ above or below 10 mmHg	(91)
	RT	51	DFS at 3 years	69	33	Median pO_2_ above or below 10 mmHg	(92)
	RT	74	DFS at 3 years	69	34	HF5 above or below 50%	(93)
	RT	106	DFS at 5 years	58	42	HF5 above or below 50%	(94)
Head and neck	RT	35	LTC at 2 years	77	33	Median pO_2_ above or below 2.5 mmHg	(96)
	RT/SG	28	DFS at 12 months	78	22	Median pO_2_ above or below 10 mmHg	(97)
	RT/CT	59	OS at 12 months	63	31	Hypoxic subvolume	(98)
	RT/CT	134	OS at 3 years	22	7	Median pO_2_ above or below 2.5 mmHg	(99)
	RT/CT/SG	310	OS at 3 years	38	28	Median pO_2_ above or below 2.5 mmHg	(100)
Sarcoma	RT/HT/SG	22	DFS at 18 months	70	35	Median pO_2_ above or below 10 mmHg	(101)
	RT/SG	28	OS at 5 years	77	28	Median pO_2_ above or below 19 mmHg	(102)
Prostate	RT	57	FFBF at 8 years	78	46	P/M ratio above or below 0.10	(103)

*^a^Patient treatments consisted of various combinations of RT (radiotherapy), CT (chemotherapy), SG (surgery), or HT (hyperthermia)*.

*^b^Response endpoints were either OS (overall survival), DFS (disease free survival), LTC (local tumor control), or FFBF (freedom from biochemical failure)*.

*^c^HF5 (percentage pO_2_ values below 5 mmHg), hypoxic subvolume (percentage pO_2_ below 5 mmHg × total tumor volume), or P/M (prostate/muscle)*.

One major focus of current cancer research is the role of cancer stem cells in tumorigenesis and therapy. Such cells amount to around 1–25% of the total viable tumor cell population (105), but they are believed to be the cells that must be completely eliminated to obtain tumor control (106). Significant effort is currently being made to identify these cells and specifically target them. However, recent evidence suggests a possible link between hypoxia and cancer stem cells (106). Hypoxia may affect cancer stem cell generation and maintenance through the upregulation of hypoxia-induced factors (105, 106). Pre-clinical studies have also shown an inverse correlation between hypoxia and local tumor control after irradiation (107, 108), suggesting that hypoxia may also actually protect the cancer stem cells from the lethal effects of radiation. If the link between cancer stem cells and hypoxia is proven, then hypoxia may be an even more important issue for radiation response than we currently believe.

### Chemotherapy

The pathophysiological characteristics of tumors play a significant role in influencing the response of the tumor cells to chemotherapy (109). An inadequate vascular supply will naturally be expected to hinder blood-borne drug delivery. A decrease in drug availability will certainly be seen in areas where flow fluctuates, especially where complete cessations in flow occur (110). In addition, the mean vascular density in tumors is lower than that found in normal tissues and, thus, diffusion distances are enlarged (1). Thus, transport of drugs from tumor microvessels to tumor cells that are distant from them will be compromised. The high IFP within solid tumors will also decrease extravasation (111). IFP at the tumor to normal tissue interface is low and as a result interstitial fluid oozes out of the tumor into the surrounding normal tissue. At the same time, it will also carry away anti-cancer drugs (37).

Cells most distant from the vascular supply will also be cycling at a reduced rate and this can act as a protective mechanism against a number of chemotherapeutic agents that work by interacting with cellular DNA and only kill the cell when it divides. Such cells are also exposed to hypoxia and acidic conditions, factors which are known to influence chemotherapeutic agent activity (111). However, these adverse microenvironmental parameters do not always have a negative effect of drug activity; some drugs are actually more effective under such conditions as illustrated in Table 3.

**Table 3:** **Influence of the hostile tumor microenvironment on the activity of chemotherapeutic drugs**.

Hypoxia dependency		pH (below 6.8) dependency	
Decreased effect	Increased effect	Decreased effect	Increased effect
Doxorubicin	Etoposide	Doxorubicin	Chlorambucil
Actinomycin D	BCNU/CCNU (?)	Daunorubicin	Melphalan
Bleomycin	Alkylating agents (?)	Bleomycin	Cyclophospamide
Vincristine	Mitomycin C	Vinblastin	Mitomycin C
Methotrexate (?)	EO9	Paclitaxol	Tiophosphamide
Cisplatin (?)	PR-104	Methotrexate	Cisplatin
5-Flurouracil (?)	TH-302	Mitoxantrone	5-Flurouracil
Procarbazine	Tirapazamine	Topotekan	Camptothecin
Streptonigrin	Banoxantrone		

*Drugs marked with (?) indicate those agents that are included in the relative categories due to their *in vitro* response, but in which *in vivo* studies suggest may not be correct. Classification is based on information from Ref. (111–114)*.

### Other Tumor Therapies

*Hyperthermia* is a less conventional therapy, but is one example where the more deficient the tumor vasculature and the more deprived the tumors cells, the better the tumor response. Blood flow, being one of the major means by which heat is dissipated from tissues, will affect the ability to heat tumors (115). Thus, the poorer the blood supply, the easier it should be to heat. This has been demonstrated *in vivo* in which blood flow was compromised using agents that could reduce tumor blood flow (116, 117). *In vitro* studies have also reported that cells under hypoxic conditions were more sensitive to killing by hyperthermia than the same cells in a well-oxygenated environment (118, 119). However, this is not a consequence of hypoxia *per se* because under well-defined nutrient conditions, acute hypoxia does not significantly alter cellular response to heat (119). However, cells under prolonged oxygen deprivation will show an increased sensitivity to heat, an effect that is the result of chronically hypoxic cells becoming acidic (118).

Another treatment modality in which the tumor microenvironment influences response is *photodynamic therapy* (PDT). It involves the administration of a photosensitizing agent and its subsequent activation by light. This reaction is strongly dependent on oxygen concentration (120–123). Cell killing by PDT appears to be complete at normal oxygen levels and above, but decreases as the oxygen concentration drops below 5% (123). This is perhaps not surprising since the mechanism of action of PDT involves the generation of singlet oxygen (124).

### Malignant Progression

One of the most striking results found with the Eppendorf electrode measurements made in cervix cancers and soft tissue sarcomas was that hypoxia influenced outcome in patients in which surgery was the primary or only treatment (90, 91, 102): this suggested that hypoxia could also influence malignant progression, especially metastatic spread. In fact, one other study in cervix was able to show that the primary tumors of patients with metastases were indeed less oxygenated than those of patients without metastases (125).

Pre-clinical studies also support the importance of hypoxia in inducing metastatic spread. The earliest studies involved exposing murine fibrosarcoma cells in culture to different oxygen concentrations for up to 24 h and then intravenously injecting these cells and observing the number of lung metastasis that develop (126). That study clearly showed that the more hypoxic the exposure, the greater the number of lung metastases. *In vivo* attempts to investigate this issue have involved either correlating the constitutive level of hypoxia in primary tumors or exposing the mice to different oxygen environments to change the level of tumor hypoxia, and then determining the number of metastases formed (127–130). The results generally show that the greater the degree of hypoxia, the more metastases observed and that acute hypoxia was better at inducing metastases than chronic. These hypoxia-induced effects on malignant progression can be the result of changes at the transcriptional level in which a range of different genes are over-expressed or at the translational level with various proteins being upregulated. Such effects can be mediated via activation of various oxygen-sensitive signaling pathways (130, 131).

Other microenvironment factors found in tumors have also been shown to influence malignant progression. Tumor cells exposed to glucose deprivation and acidosis prior to intravenous injection into mice resulted in more lung metastases than cells not exposed to these conditions (132, 133). The deleterious effects of glucose deprivation *in vivo* have made it impossible to investigate its potential effects on malignancy in animals, but there have been attempts to relate pHe *in vivo* with metastases formation. Those studies involved either experimentally increasing tumor acidity (134) or simply making probe measurements of pHe in untreated tumors (135). But in both situations, no correlations were found between pH and metastases. This is perhaps somewhat surprising because one of the major factors causing acidity is lactate production and clinical studies have shown high tumor lactate concentrations to be associated with an increased risk of nodal and distant metastases (49), and a shorter overall and disease-free survival (50).

The relationship between IFP and metastatic dissemination is also controversial. Using melanoma xenografts, an association was found between high IFP and the development of pulmonary and lymph node metastases (136). Another study from the same research group reported that IFP was significantly higher in intramuscularly implanted TS-415 cervix carcinoma xenografts that metastasized than those that did not (135). However, that same study showed this not to be the case for CK-160 cervical xenografts. Furthermore, a lack of any correlation between IFP and metastatic spread was also reported using two other cervical xenograft models (ME180 and SiHa) regardless of whether they were grown intramuscularly or orthotopically (137). Clinical studies have been less controversial, with IFP measurements in cervix cancer patients being shown to predict for survival (41) and for both nodal and distant metastases (94). A possible relationship between IFP and metastatic disease in other tumor sites is not yet known.

## Conclusion

The pathophysiological characteristics of the tumor microenvironment are very different from those conditions found in normal tissues. In many respects, the tumor microenvironment can be considered abnormal and hostile. These adverse pathophysiological conditions, especially hypoxia, are now known to play a significant role in determining the tumor response to therapy and influencing the metastatic potential of tumors. Clearly, the future requirement is the application of methods by which one can accurately and reliably image the various important microenvironmental parameters, especially using techniques that are routinely available in the clinic. With such information, it should be possible to identify those patients, on an individual basis, that would be expected to have a poor prognosis and, thus, select appropriate additional treatments to dramatically improve that prognosis.

## Author Contributions

Both authors contributed equally to the concept, development, and the writing of this manuscript.

## Conflict of Interest Statement

The authors declare that the research was conducted in the absence of any commercial or financial relationships that could be construed as a potential conflict of interest.
